# A call to strengthen data in response to COVID-19 and beyond

**DOI:** 10.1093/jamia/ocaa308

**Published:** 2020-12-22

**Authors:** Natasha Azzopardi-Muscat, Hans Henri P Kluge, Samira Asma, David Novillo-Ortiz

**Affiliations:** 1 Regional Office for Europe, World Health Organization, Copenhagen, Denmark; 2 Division of Data, Analytics and Delivery for Impact, World Health Organization, Geneva, Switzerland

**Keywords:** Data, health information systems, COVID-19

## Abstract

The COVID-19 (coronavirus disease 2019) pandemic has underscored the critical need for all countries to strengthen their health data and information systems and ensure the routes the data travel, from submission to use, are unobstructed. Timely, credible, reliable, and actionable data are key to ensuring that political decisions are data driven and facilitate understanding, monitoring, and forecasting. To ensure that critical decisions related to the wider health and socioeconomic effects of this pandemic are data driven, each country needs to develop or enhance a national data governance plan that includes a clear coordination mechanism, well-defined and documented data processes (manual or electronic), the exchange of data, and a data culture to empower users. In addition, countries should now more than ever invest and enhance their data and health information systems to ensure that all decisions are data driven and that they are prepared for what is next.

The COVID-19 (coronavirus disease 2019) pandemic has underscored the critical need for all countries to strengthen their health data and information systems and ensure the routes the data travel, from submission to use, are unobstructed. This applies widely to systems even beyond the health sector. In particular, the inability to effectively leverage the volume and different types of available data from routine health information systems has been a notable shortcoming of the pandemic response to date. The main barriers result from the lack of health data standardization, such as the definitions, calculations, and formats of the data; delays in receiving data; lack of integration and interoperability between the different data and health information systems; and the lack of trained people to manage and use these data. All these problems have coexisted for decades but only now have simultaneously impacted all countries and provoked an unprecedented global crisis.

Timely, credible, reliable, and actionable data are key to ensure that political decisions are data driven and facilitate understanding, monitoring, and forecasting. Furthermore, the combination of disaggregated data, nonclassical datasets (demographics, genetics, social and family history, lifestyle, socioeconomics, and environment), and big data solutions can help to reveal patterns and enable precise targeting of decisions and measures. In this comment, we emphasize the imperative for each country to overcome these barriers so that data gaps can be closed. Here, we summarize main actions that can be taken to ensure that critical decisions related to the wider health and socioeconomic effects of this pandemic, as well as future health challenges, are data-driven.

First, to facilitate national and global data comparison and to facilitate the secondary use of data, a national data coordination mechanism must be established. Through an appropriate suite of indicators and a data governance framework that includes law enforcement systems, this mechanism can effectively coordinate and centralize different data providers across the country and standardize data elements (eg, definitions, the reporting process, calculations, visualizations). This mechanism can then collect, manage, and disseminate consistent and complete data necessary for policy decision making in a public health emergency.

Furthermore, the monitoring of the public health environment and resources needed to tackle a public health threat must be enhanced. To do so, each data provider must clearly define and document procedures—manual or electronic—on data flows and processes that can result in a rapid detection of threats and needs, and then facilitate a fast and effective response. In addition, where digital data infrastructure exists, digital solutions, such as big data or artificial intelligence, can contribute to rapid disease prevention, automation of processes, and more efficient resource management.

When these two actions have been taken, countries will be able to effectively exchange relevant data that will inform public health and socioeconomic decision makers so that they can respond responsibly to any public health threat. Adopting the same definitions and data standards within a country, with the support of appropriate digital infrastructures, will facilitate the integration and interoperability of the different data and health information systems across a country. This in turn will further enable effective global and reliable comparability, as promoted and agreed on by all countries included in the International Health Regulations.^1^

Finally, countries should promote a data-driven culture focusing on empowering citizens to control the data they produce and develop their data skillsets. We live in a data and digitalized ecosystem that requires new solutions and new skills to properly address day-to-day events that require informed decisions. All people need to understand their rights as well as the benefits and risks of sharing data, especially related to ethics, privacy, and security. In addition, those involved as policy decision makers, from data production to data use, must develop the necessary skills to access and manage the right data, at the right time and for the right purpose.

Each country needs to develop or enhance a national data governance plan that includes a clear coordination mechanism, well-defined and documented data processes (manual or electronic), the exchange of data, and a data culture to empower users.

So far, along with the immense work that health workers are carrying out around the world, data are a critically important tool to effectively tackle this pandemic. Countries should more than ever invest and enhance their data and health information systems to ensure that all decisions are data driven and that they are prepared for what is next. Member States can count on the support of the World Health Organization to help them strengthen their health information systems, as highlighted in the 13th General Programme of Work 2019-2023, as well as its aligned, tailored workplan for the European region, the European Programme of Work 2020-2025. Working together, these goals can be achieved.

## AUTHOR CONTRIBUTIONS

All authors contributed sufficiently and meaningfully to the conception, design, drafting, editing, and revising the manuscript. All authors approved the final version for submission and agree to be accountable for all aspects of the work.

## CONFLICT OF INTEREST STATEMENT

The authors have no competing interests to declare.
